# Pan-Cancer and Single-Cell Analysis Reveals CENPL as a Cancer Prognosis and Immune Infiltration-Related Biomarker

**DOI:** 10.3389/fimmu.2022.916594

**Published:** 2022-06-30

**Authors:** Ziyang Feng, Yu Chen, Changjing Cai, Jun Tan, Ping Liu, Yihong Chen, Hong Shen, Shan Zeng, Ying Han

**Affiliations:** ^1^ Department of Oncology, Xiangya Hospital, Central South University, Changsha, China; ^2^ Key Laboratory for Molecular Radiation Oncology of Hunan Province, Xiangya Hospital, Central South University, Changsha, China; ^3^ Department of Neurosurgery, Xiangya Hospital, Central South University, Changsha, China; ^4^ National Clinical Research Center for Geriatric Disorders, Xiangya Hospital, Central South University, Changsha, China

**Keywords:** pan-cancer, CENPL, immune infiltration, LUAD, G0/G1 arrest, apoptosis

## Abstract

**Background:**

Centromere protein L (CENPL) is an important member of the centromere protein (CENP) family. However, the correlation between CENPL expression and cancer development and immune infiltration has rarely been studied. Here, we studied the role of CENPL in pan-cancer and further verified the results in lung adenocarcinoma (LUAD) through *in vitro* experiments.

**Methods:**

The CENPL expression level was studied with TIMER 2.0 and Oncomine databases. The potential value of CENPL as a diagnostic and prognostic biomarker in pan-cancer was evaluated with the TCGA database and GEPIA. The CENPL mutation character was analyzed using the cBioPortal database. The LinkedOmics and CancerSEA databases were used to carry out the function analysis of CENPL. The role of CENPL in immune infiltration was studied using the TIMER and TISIDB websites. Moreover, the expression of CENPL was detected through RT-qPCR and Western blotting. Immunohistochemistry was used to evaluate the infiltration level of CD8^+^ T cells. Cell proliferation was detected by EdU and CCK8. A flow cytometer was used to analyze the influence of CENPL in cell cycle and apoptosis.

**Results:**

CENPL was increased in most of the cancers. The upregulation and mutation of CENPL were associated with a poorer prognosis in many cancers. The results showed a significant positive correlation between CENPL and myeloid-derived suppressor cell (MDSC) infiltration and a negative correlation between CENPL and T-cell NK infiltration in most of the cancers. CENPL regulated cell proliferation and cell cycle, and was negatively correlated with the inflammation level of LUAD. The *in vitro* experiments suggested that CENPL was increased in LUAD tissue and cell lines. There was a negative correlation between CENPL expression and CD8^+^ T-cell infiltration. The knockdown of CENPL significantly suppressed the expression of CDK2 and CCNE2, and induced G0/G1 arrest and apoptosis of LUAD.

**Conclusions:**

CENPL may function as a potential biomarker and oncogene in pan-cancer, especially LUAD. Furthermore, CENPL was associated with immune cell infiltration in pan-cancer, providing a potential immune therapy target for tumor treatment.

## Introduction

Nowadays, cancer has become a major threat to public health. Although huge improvements have been made in cancer treatment, including immunotherapy, target therapy, and radiotherapy, patients’ 5-year overall survival (OS) remains unsatisfactory (1). In recent years, immunotherapy has made huge success in cancer treatment. For example, the immune checkpoint inhibitor has achieved great success in the clinic (2–4). Moreover, with the rapid development of the TCGA database and the GEO database, it is more convenient to further analyze the correlation and impact of a single gene on cancer prognosis and immune infiltration. Thus, it is necessary to find a novel diagnostic and prognostic biomarker for cancer. Furthermore, the huge success of immunotherapy makes the immune-related biomarker even more important.

Centromere protein L (CENPL) is a member of the centromere protein (CENP) family and plays significant roles in mediating cell division (5, 6). CENPs are important members of the centromere and kinetochore, which determine the separation of chromosomes during mitosis and meiosis. Many CENPs have been currently identified, including CENPA, CENPH, CENPI, and CENPU (7–11). Among them, CENPA plays a significant role in cancer. For example, the upregulation of the CENPA/Myc/Bcl2 axis significantly enhances the sensitivity of retinoblastoma cells to cisplatin (12). Furthermore, CENPA may function as a prognostic biomarker in lung adenocarcinoma (LUAD) (13). However, the role of CENPL in cancer remains fragmentary.

Thus, we conducted a comprehensive analysis and assessed the potential value of CENPL in cancer diagnosis and prognosis. Moreover, we also performed enrichment analysis of CENPL co-expression genes and studied its association with immune infiltration. Furthermore, we verified our bioinformatics results through *in vitro* experiments. All in all, our results show that CENPL can function as an oncogene and immune infiltration-related biomarker in pan-cancer, especially LUAD.

## Materials and Methods

### Data Collection

TCGA transcriptome RNA-seq data were downloaded using the Broad Institute platform (https://gdac.broadinstitute.org/).

### Analysis of CENPL mRNA Expression

The Oncomine database (oncomine.org) and the TIMER 2.0 database (timer.cistrome.org) were used to evaluate the mRNA expression level of CENPL. At first, we searched “CENPL” in the Oncomine database, and the p-values < 0.05 and folding change < 1.5 were considered significant. Next, in the TIMER 2.0 database, the “Gene_DE” module was used to analyze the mRNA expression level of CENPL. The association between CENPL and cancer stage was analyzed with the “Stage plots” module of GEPIA (gepia2.cancer-pku.cn/#index).

### Diagnostic and Prognostic Analysis

The potential value of CENPL in cancer diagnosis was detected with ROC curve with the data from the TCGA database. AUC > 0.85 was thought as a high diagnostic value. The potential value of CENPL in cancer prognosis was analyzed via OS and disease-free survival (DFS) from the GEPIA database.

### Mutation Character Analysis

The mutation character of CENPL in different cancers was analyzed with the cBioPortal tool (http://www.cbioportal.org/). “TCGA Pan Cancer Atlas Studies” was selected as the cohort. Then, we entered “CENPL” in the “Query” module. CENPL alteration sites, types, and numbers can be found in the “cancer type summary” and “mutation” module. The correlation between CENPL mutation and the clinical prognosis was obtained from the “comparison/survival” module.

### The Function and Enrichment Analysis

Gene co-expression analysis of CENPL was performed using LinkedOmics (www.linkedomics.org/login.php). The “HiSeq RNA” platform and “TCGA_LUAD” cohort were selected for the analysis. Pearson test was used to detect the correlation between CENPL and the co-expression genes.

The correlation between CENPL and 14 cancer functional states was analyzed using single-cell sequence data from the “correlation plot” module of the CancerSEA website (biocc.hrbmu.edu.cn/CancerSEA/home.jsp).

### Immune Infiltration Analysis

The “GENE” module of the TIMER database was used to evaluate the infiltration level of immune cells in 32 cancer types. TISIDB (cis.hku.hk/TISIDB/index.php) was used to analyze the correlation between CENPL and major histocompatibility complexes (MHCs) and chemokine receptors.

### Cell Culture

Beas2B (normal pulmonary epithelial cell), PC9, H1975, A549, and H1437 (human lung cancer cell lines) were derived from the Institutes of Biomedical Sciences. Cells were cultured at 37°C with 10% FBS and 1% penicillin/streptomycin in RPMI-1640.

### Specimen Collection

LUAD tissues were collected from Xiangya Hospital. The specimen was stored at −80°C immediately after the surgery. All the patients received a LUAD diagnosis with the results of the histopathological examination. The studies were approved by Ethics Committees of Xiangya Hospital. The patients’ clinicopathological information can be found in **Table 1**.

**Table 1:** Clinicopathological parameters of LUAD cohort in Xiangya Hospital.

Patient NO.	Age (y)	Gender	History of smoking	History of alcohol	Location in lung	Differentiaion	T classification	N classification	M classification	TNM stage	Living status and time(days)	CENPL relative expression
1	60	Female	No	No	Right upper	Moderately	1a	2	0	IIIA	Living/2024	2.757631102
2	55	Male	No	No	Right upper	Poorly	1	0	0	IA	Living/1759	2.697716191
3	66	Male	Yes	No	Right lower	Poorly	1b	0	0	IA	Living/1457	0.244443329
4	41	Female	No	No	Left lower	Well	2a	0	0	IA	Living/1291	0.11338954
5	67	Male	Yes	No	Right upper	/	2	2	0	IIIA	Dead/1266	0.121970052
6	65	Male	Yes	No	Left upper	Poorly	2a	0	0	IA	Living/1268	2.671662348
7	60	Male	Yes	No	Left upper	Moderately	2	2	0	IIIA	Dead/748	0.625218754
8	59	Female	/	/	Left upper	/	2	0	0	IB	Living/1126	0.650366834
9	58	Female	No	No	Right upper	Well	1b	2	0	IIIA	Living/1643	4.910346156
10	53	Male	Yes	Yes	Right lower	Poorly	2c	0	0	IIA	Dead/615	0.789007838
11	69	Male	Yes	Yes	Right upper	Poorly	3	0	0	IIB	Living/1285	0.135412198
12	63	Male	Yes	Yes	Right upper	Well-Moderately	1c	2	0	IIIA	Living/1399	7.193870084
13	36	Male	No	No	Left upper	/	1c	2	0	IIIA	Living/1338	0.152539373
14	45	Female	/	No	Right lower	/	0	0	1	IV	Living/62	3.995745298
15	61	Male	No	Yes	Right lower	Poorly	1	2	0	IIIA	Dead/1491	0.088769722
16	53	Male	Yes	/	Left lower	Moderately	2	0	0	IB	Living/1674	6.459017468
17	53	Male	Yes	No	Left upper	Moderately-Poorly	1b	2	0	IIIA	Living/409	2.946329291
18	66	Male	Yes	Yes	Left upper	/	2	0	0	IB	Dead/1113	0.732881534
19	73	Male	Yes	Yes	Right upper	Poorly	1	0	0	IA	Living/1552	1.334120069
20	60	Male	Yes	No	Left lower	Well-Moderately	1	0	0	IA	Living/1491	0.908902748
21	59	Male	/	No	Left upper	Moderately-Poorly	2	0	0	IB	Dead/1583	2.164688313
22	35	Female	No	No	Right lower	Well	4	2	0	IIIB	Living/891	0.357811515
23	74	Male	Yes	Yes	Right lower	Moderately-Poorly	4	0	0	IIIA	Living/335	0.618514161
24	58	Female	/	No	Right upper	Moderately	2	0	0	IB	Living/624	1.528012423
25	52	Male	Yes	No	Right lower	Poorly	1	2	0	IIIA	Dead/1552	0.023937207
26	60	Male	No	Yes	Right upper	Moderately	1	0	0	IA	Living/1430	0.14419824
27	32	Female	No	No	Left lower	Moderately	3	3	0	IIIC	Dead/1399	0.016709341
28	42	Male	Yes	Yes	Right upper	Moderately-Poorly	1	0	0	IA	Dead/1369	11.53368936
29	60	Female	No	Yes	Right lower	Poorly	2	2	0	IIIA	Living/1004	1.344631759

### Real-Time Quantitative PCR

The RNA was separated with Trizol reagent, and PrimeScript™ Kit was used to perform the reverse transcription. SYBR Green assay was used to carry out the RT-qPCR reaction. The primers were as follows: CENPL: F: CTGGCTGGTTCTGCTGTGTA; R: GGCAGCCATCCAGGAAAGAT. GAPDH: F: TGTGGGCATCAATGGATTTGG; R: ACACCATGTATTCCGGGTCAAT. 2−ΔΔCt values were calculated to perform the quantitative analysis.

### Western Blotting

The total protein was extracted using RIPA buffer with phosphatase inhibitor and protease inhibitor. After separating by SDS-PAGE, the protein was transferred onto polyvinylidene fluoride membranes under 250 mA for 100 min. Five percent skim milk was used to block the membrane. Next, the membrane was incubated with primary antibodies at 4°C for 12 h. Subsequently, the membrane was incubated with the secondary antibody at 37°C for 60 min. After washing 3 times with TBST, the signal was detected using the ChemiDocXRS+ System. Finally, Image Lab software was used for the quantitative analysis of protein.

### Immunohistochemistry Procedure

Tumor tissue sections were deparaffinized using xylene and rehydrated with ethanol. Next, these sections were boiled by microwaving with citrate for 10 min. After eliminating endogenous peroxidase activity with 3% H2O2, the slides were incubated with anti-CD8 antibody (ab101500, Abcam) at 1:100 overnight at 4°C. Next, the slides were incubated using secondary antibody at 25°C for 60 min. After staining with DAB and hematoxylin, the CD8+ T cells were calculated using a microscope.

### Cell Proliferation Assays

A total of 4,000 cells were seeded into 96-well plates. Subsequently, 10 μl of the CCK-8 was mixed to every well. The absorbance at 450 nm was measured after 2.5 h.

### EdU Assay

A total of 3,000 cells were seeded into 96-well plates. Next, the cells were maintained with 20 μM EdU for 3 h and fixed with 4% paraformaldehyde. Subsequently, the cells were treated with 100 μl of Apollo solution for 30 min. After washing with 0.5% Triton X-100 and staining with DAPI solution, the image was captured with a microscope.

### Cell Cycle Test

A total of 100,000 cells were seeded in each well of 6-well plates. The cells were collected and treated with 1 ml of DNA Staining solution and 10 μl of Permeabilization solution for 30 min. Subsequently, the sample was detected using a flow cytometer right away.

### Apoptosis Test

A total of 100,000 cells were seeded into 6-well plates. Next, the cells were collected (including the cells in medium) and treated with 5 μl of Annexin V-APC and 10 μl of 7-AAD for 5 min. Subsequently, the sample was detected using a flow cytometer right away.

### Statistical Analysis

The differences among groups were detected using t-test. The correlation analysis was evaluated with Spearman’s test. The analysis was performed with SPSS 17.0 and p-value < 0.05 was considered significant.

## Results

### CENPL Was Upregulated in Multiple Human Cancers

**Figure 1:**
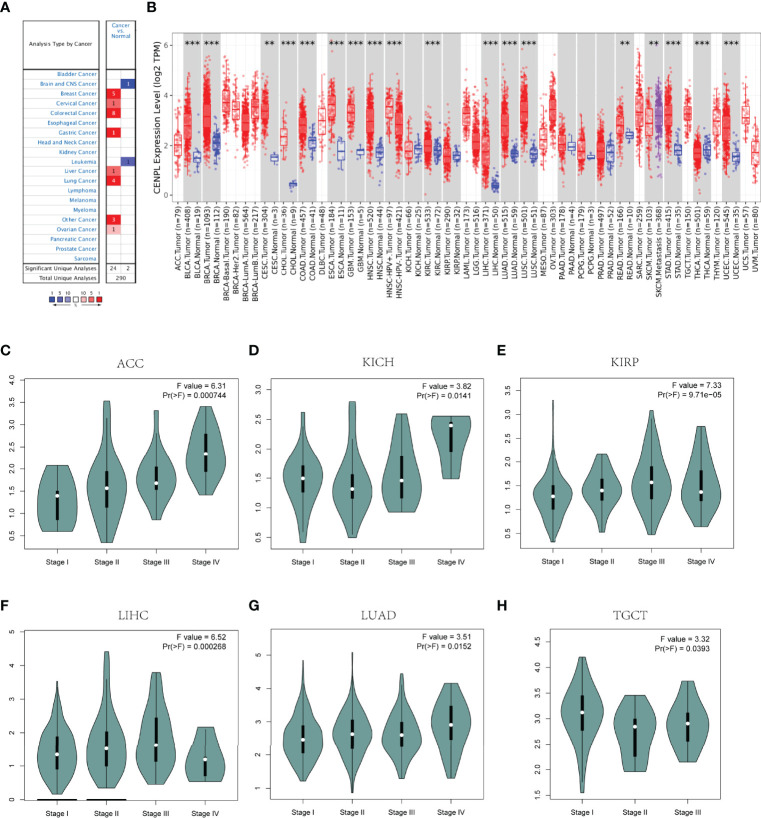
CENPL expression profiles in pan-cancer. **(A)** The expression of CENPL in the Oncomine database. **(B)** The expression of CENPL in the TIMER 2.0 database. **(C–H)** The correlation between CENPL expression and cancer stage (**p < 0.01, ***p < 0.001).

At first, Oncomine was used to study the expression level of CENPL. The results obtained show that CENPL was upregulated in breast cancer, lung cancer, colorectal cancer, gastric cancer, liver cancer, cervical cancer, and ovarian cancer (**Figure 1A**). By contrast, CENPL was downregulated in the brain and CNS cancer and leukemia (**Figure 1A**). To further explore the expression level of CENPL, we analyzed CENPL expression in the TCGA datasets using the TIMER 2.0 database. The results obtained show that CENPL was upregulated in most of the cancers (**Figure 1B**), including bladder urothelial carcinoma (BLCA), breast invasive cancer (BRCA), cervical and endocervical cancer (CESC), cholangiocarcinoma (CHOL), colon adenocarcinoma (COAD), esophageal carcinoma (ESCA), glioblastoma multiforme (GBM), head and neck squamous cell carcinoma (HNSC), kidney renal clear cell carcinoma (KIRC), liver hepatocellular carcinoma (LIHC), lung adenocarcinoma (LUAD), lung squamous cell carcinoma (LUSC), stomach adenocarcinoma (STAD), and uterine corpus endometrial carcinoma (UCEC). CENPL was upregulated in metastatic tumor of skin cutaneous melanoma compared with primary tumor. However, CENPL was downregulated in thyroid carcinoma (THCA).

Next, we analyzed the association between CENPL and the stages of pan-cancers in the GEPIA2 website. CENPL was associated with the stage of kidney renal papillary cell carcinoma (KIRP), adrenocortical carcinoma (ACC), LIHC, kidney chromophobe (KICH), LUAD, and testicular germ cell tumors (TGCT) (**Figures 1C–H**).

### The Diagnostic and Prognostic Value of CENPL in Pan-Cancer

**Figure 2:**
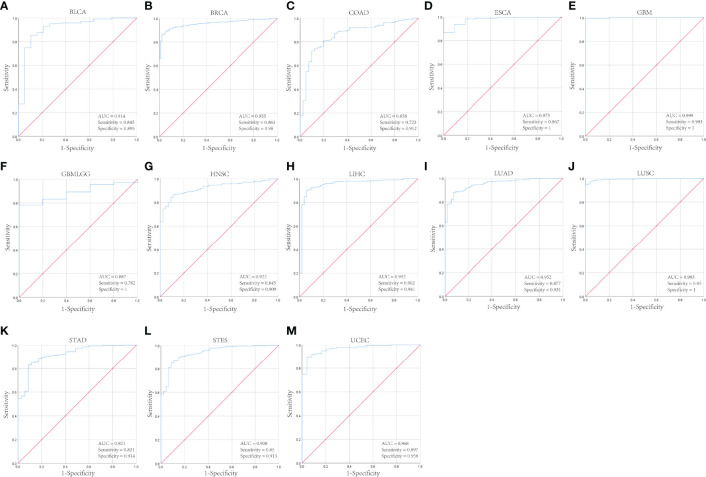
The diagnostic value of CENPL in pan-cancer. **(A–M)**. The ROC curves of CENPL in BLCA, BRCA, COAD, ESCA, GBM, GBMLGG, HNSC, LIHC, LUAD, LUSC, STAD, STES, UCEC.

Then we analyzed the diagnostic value of CENPL in various cancers using the ROC curve. As shown in **Figures 2A–M**, CENPL may act as a perfect diagnostic marker in BLCA (AUC = 0.914), BRCA (AUC = 0.955), CAOD (AUC = 0.858), ESCA (AUC = 0.975), GBM (AUC = 0.999), glioma (GBMLGG; AUC = 0.887), HNSC (AUC = 0.922), LIHC (AUC = 0.952), LUAD (AUC = 0.952), LUSC (AUC = 0.993), STAD (AUC = 0.921), stomach and esophageal carcinoma (STES; AUC = 0.938), and UCEC (AUC = 0.968).

**Figure 3:**
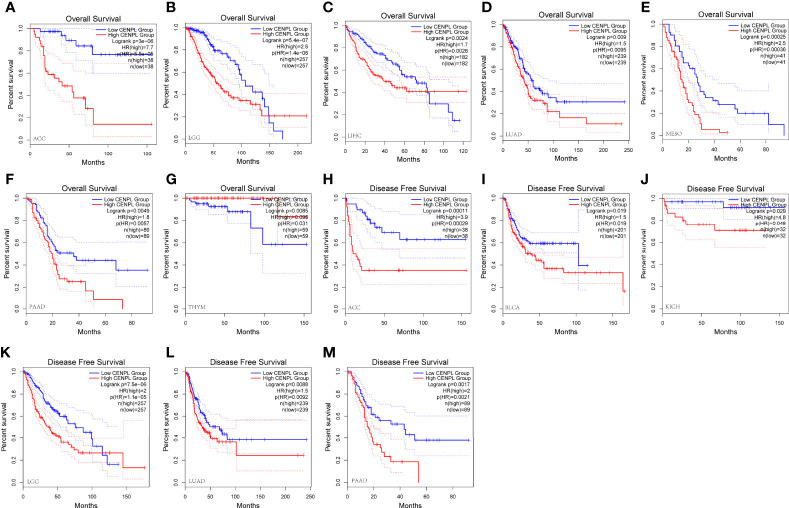
The prognosis value of CENPL in pan-cancer. **(A–G)** Kaplan–Meier analysis of the association between CENPL expression and OS. **(H–M)** Kaplan–Meier analysis of the association between CENPL expression and DFS.

Furthermore, increased expression of CENPL was linked to poor OS in LGG (p = 5.4e-07), ACC (p = 3e-06), MESO (p = 0.00025), PAAD (p = 00049), LIHC (p = 0.0024), and LUAD (p = 0.009) (**Figures 3A–F**). By contrast, increased expression of CENPL was linked to better OS in THYM (p = 0.0085) (**Figure 3G**). DFS results suggested that higher CENPL expression was correlated with poorer prognosis in LGG (p = 7.5e-06), ACC (p = 0.00011), PAAD (p = 0.0017), LUAD (p = 0.0088), BLCA (p = 0.019), and KICH (p = 0.029) (**Figures 3H–M**).

### The Characteristics of CENPL Mutations in the TCGA Pan-Cancer Cohort

**Figure 4:**
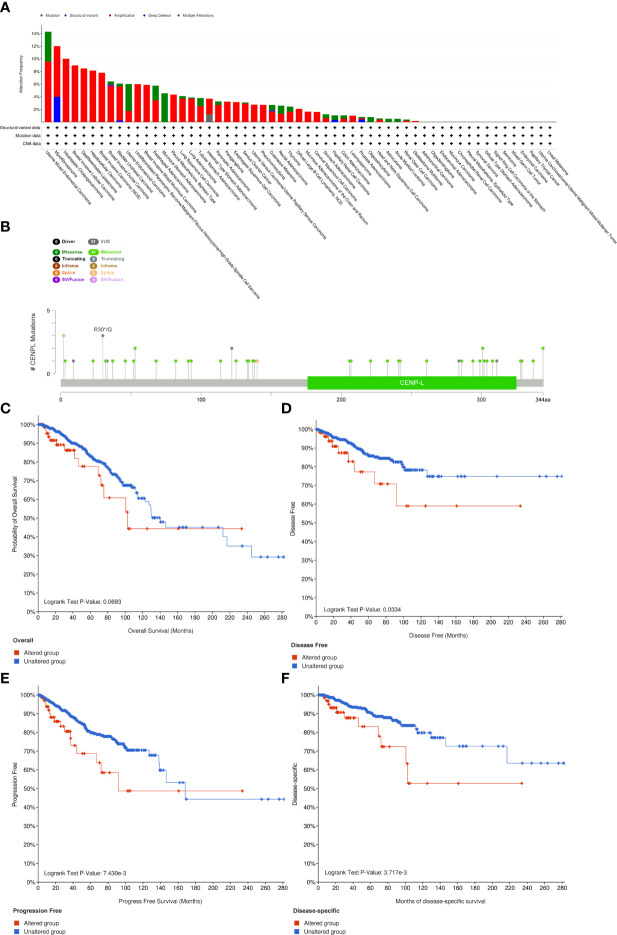
The mutation character of CENPL in pan-cancer. **(A)** Alteration frequency of CENPL in pan-cancer. **(B)** The subtypes and distributions of CENPL somatic mutations. **(C–F)** OS, DFS, PFS, and DSS analysis stratified by CENPL mutation status in breast invasive carcinoma.

Next, we analyzed the CENPL alteration status of the TCGA cohorts. As shown in **Figure 4A**, there were 42 kinds of cancers with CENPL alteration and uterine mixed endometrial carcinoma had the highest frequency of 14.29%. The CENPL alteration sites, types, and numbers are further presented in **Figure 4B**. Then, we further analyzed the correlation between CENPL mutation and the clinical prognosis. The data of **Figures 4C–F** suggested that patients that had breast invasive carcinoma with CENPL mutation had worse prognosis in DFS (p = 0.0334), DSS (p = 3.717e-3), and PFS (p = 7.430e-3), but not OS (p = 0.0693).

### CENPL Is Related to Immune Infiltration in Pan-Cancer

**Figure 5:**
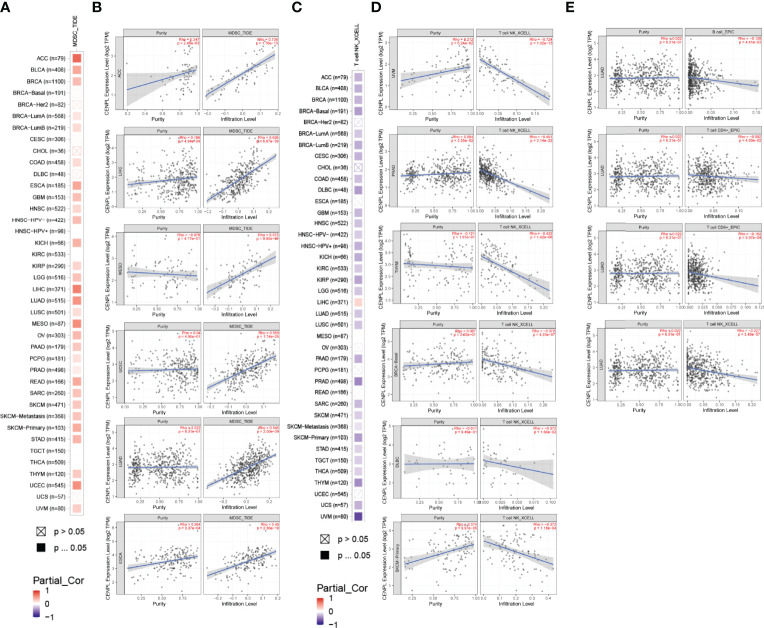
The association between CENPL expression and immune cell infiltration. **(A, B)** CENPL expression is positively associated with MDSC infiltration in pan-cancer. **(C, D)** CENPL expression is negatively associated with NKT cell infiltration in pan-cancer. **(E)** CENPL expression is negatively associated with the infiltration level of B cells, CD4^+^ T cells, CD8^+^ T cells, and NKT cells.

TIMER 2.0 was used to explore the potential role of CENPL in immune cell infiltration. The results suggested a significant positive correlation between myeloid-derived suppressor cell (MDSC) infiltration level and CENPL expression in most of the tumors (**Figure 5A**); the top 6 tumors were ACC (rho = 0.736, p = 1.16e-13), LIHC (rho = 0.626, p = 6.67e-39), MESO (rho = 0.573, p = 9.80e-09), UCEC (rho = 0.559, p = 1.74e-25), LUAD (rho = 0.545, p = 2.00e-39), and ESCA (rho = 0.45, p = 2.36e-10) (**Figure 5B**). Interestingly, the results suggested a negative association between the infiltration level of T-cell NK and CENPL expression in most of the tumors (**Figure 5C**); the top 6 tumors were UVM (rho = −0.724, p = 1.02e-13), PRAD (rho = −0.451, p = 3.14e-22), THYM (rho = −0.432, p = 1.42e-06), BRCA-Basal (rho = −0.372, p = 4.23e-07), DLBC (rho = −0.372, p = 1.66e-02), and SKCM-Primary (rho = −0.372, p = 1.18e-04) (**Figure 5D**). Finally, we studied the role of CENPL in immune cell infiltration of LUAD. There was a negative association between CENPL and the infiltration level of B cells (rho = −0.128, p = 4.41e-03), CD4+ T cells (rho = −0.092, p = 4.03e-02), CD8+ T cells (rho = −0.162, p = 3.07e-04), and T-cell NK (rho = −0.227, p = 3.48e-07) (**Figure 5E**).

**Figure 6:**
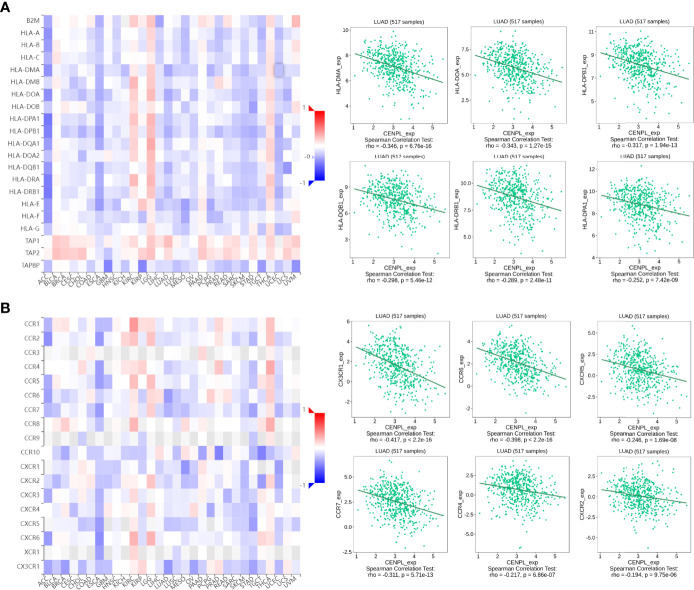
The correlation between CENPL expression and major histocompatibility complexes (MHCs) and chemokine receptors in the TISIDB database. **(A)** The expression of CENPL is negatively associated with most MHCs in pan-cancer. **(B)** The expression of CENPL is negatively associated with most chemokine receptors in pan-cancer.

Next, we further analyzed the association between CENPL and MHCs and chemokine receptors in the TISIDB database. Our results suggested that the CENPL expression is negatively associated with most of the MHCs (**Figure 6A**). As for LUAD, the top 6 MHCs were HLA-MA (rho = −0.346, p = 6.76e-16), HLA-DOA (rho = −0.343, p = 1.27e-15), HLA-DPB1 (rho = −0.317, p = 1.94e-13), HLA-DQB1 (rho = −0.298, p = 5.46e-12), HLA-DRB1 (rho = −0.298, p = 2.48e-11), and HLA-DPA1 (rho = −0.252, p = 7.42e-09). **Figure 6B** shows the correlations between CENPL expression and 18 kinds of chemokine receptors. The results suggested that CENPL was negatively associated with many chemokine receptors in LUAD; the top 6 were CX3CR1 (rho = −0.417, p < 2.2e-16), CCR (rho = −0.398, p < 2.2e-16), CXCR5 (rho = −0.246, p = 1.69e-08), CCR7 (rho = −0.311, p = 5.71e-13), CCR4 (rho = −0.217, p = 6.86e-07), and CXCR2 (rho = −0.194, p = 9.75e-06).

### The Function Analysis of CENPL in LUAD

**Figure 7:**
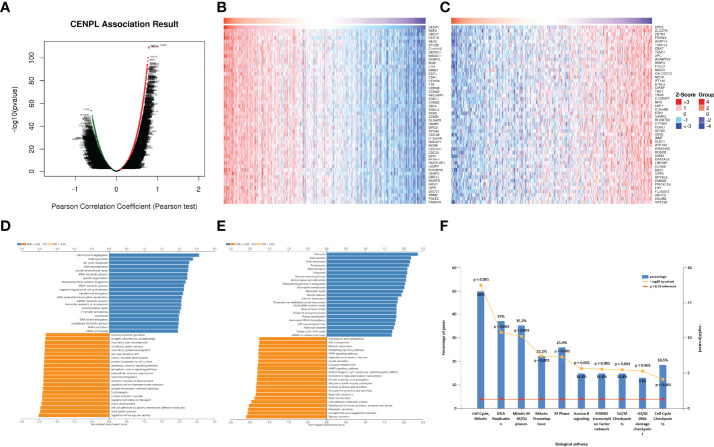
The enrichment analysis of CENPL co-expression genes in LUAD. **(A)** The CENPL co-expression genes in LUAD. **(B, C)** The top 50 genes positively and negatively correlated to CENPL. **(D, E)** GO and KEGG analysis of CENPL co-expression genes in the LUAD cohort. **(F)** The biological pathway analysis of CENPL co-expression genes in the LUAD cohort.

Then, we chose LUAD to further explore CENPL’s biological function in LinkedOmics database. **Figure 7A** suggests the positively and negatively related genes with CENPL. The top 50 genes are shown in **Figures 7B, C**. Moreover, GO analysis (Biological function) demonstrated that CENPL mainly joins in chromosome segregation, DNA replication, cell cycle checkpoint, DNA recombination, double-strand break repair, tRNA metabolic process, spindle organization, ribonucleoprotein complex biogenesis, etc. (**Figure 7D**). KEGG analysis illustrated enrichment in cell cycle, spliceosome, DNA replication, proteasome, RNA transport, ribosome, Fanconi anemia pathway, etc. (**Figure 7E**). Biological pathway analysis showed that CENPL may participate in cell cycle, mitotic, DNA replication, mitotic M-M/G1 phase, mitotic prometaphase, M phase, aurora B signaling, FOXM1 transcription factor network, G2/M checkpoints, G2/M DNA damage checkpoints, and cell cycle checkpoints (**Figure 7F**).

**Figure 8:**
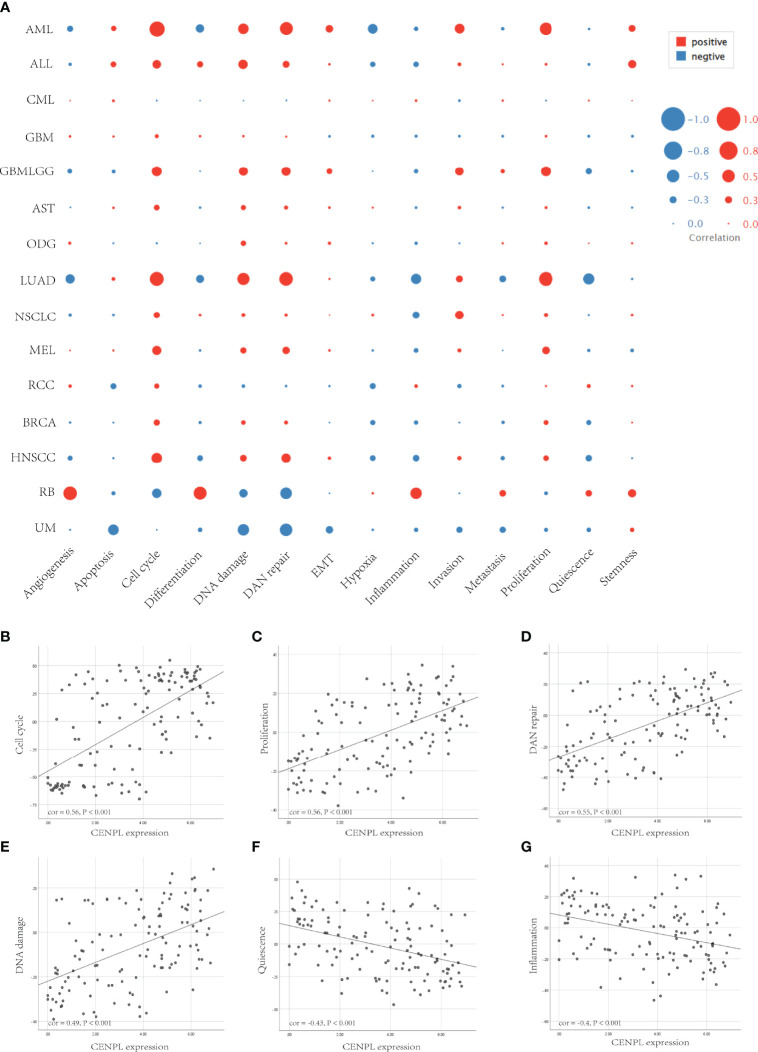
The correlation between CENPL expression and 14 cancer functional states using single-cell sequence data from the CancerSEA database. **(A)** The correlation between CENPL expression and 14 cancer functional states in pan-cancer. **(B–E)** The expression of CENPL is positively correlated to the cell cycle, proliferation, DNA repair, and DNA damage of LUAD. **(F, G)** The expression of CENPL is negatively correlated to the quiescence and inflammation of LUAD.

Next, we further analyzed the correlation between CENPL and 14 cancer functional states using single-cell sequence data of CancerSEA. CENPL was positively associated with cell cycle in most of the tumors (**Figure 8A**). Interestingly, CENPL was negatively associated with the inflammation status of most of the tumors, and LUAD was the top 1 (**Figure 8A**). As for LUAD, there is a significant positive correlation between CENPL expression and cell cycle (cor = 0.56), proliferation (cor = 0.56), DNA repair (cor = 0.55), and DNA damage (cor = 0.49), and a negative correlation with quiescence (cor = −0.43) and inflammation (cor = −0.4) (**Figures 8B–G**).

### Knockdown of CENPL Induced Apoptosis and G0/G1 Arrest of LUAD

**Figure 9:**
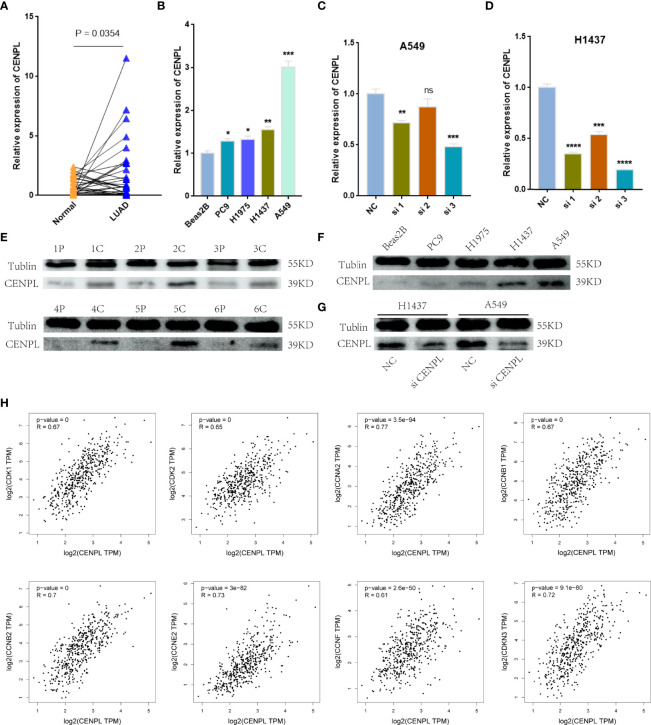
Validation of CENPL expression. **(A, B)** CENPL mRNA expression in LUAD tissues and cell lines. **(C, D)** The knockdown efficiency of CENPL mRNA in A549 and H1437 cells. **(E, F)** CENPL protein expression in LUAD tissues and cell lines. **(G)** The knockdown efficiency of CENPL protein in A549 and H1437 cells. **(H)** The correlation between CENPL expression and CDK1, CDK2, CCNA2, CCNB1, CCNB2, CCNE2, CCNF, and CDKN3 in the GEPIA2.0 database (*p < 0.05, **p < 0.01, ***p < 0.001, ****p < 0.0001, ns: no significance).

Next, we further confirm our bioinformatics results through *in vitro* experiments. CENPLs were significantly upregulated in LUAD tissues (**Figures 9A, E**) (C: cancer P: paracancerous non-cancer). Furthermore, CENPL was significantly upregulated in lung cancer cell lines as well (**Figures 9B, F**).

**Figure 10:**
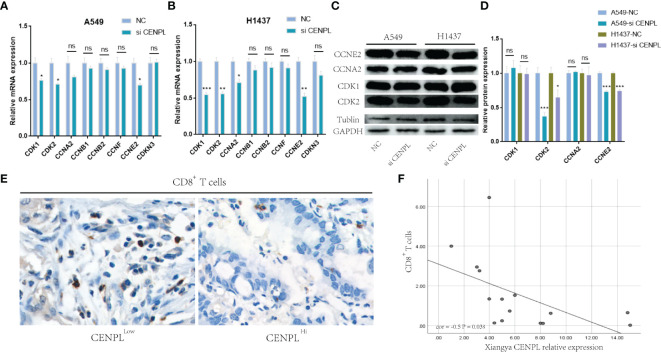
Knockdown of CENPL significantly suppressed the expression of CDK2 and CCNE2. **(A, B)** Knockdown of CENPL suppressed the mRNA expression of CDK1, CDK2, CCNA2, and CCNE2 in A549 and H1437 cells. **(C, D)** Knockdown of CENPL significantly suppressed the protein expression of CDK2 and CCNE2 in A549 and H1437 cells. **(E, F)** CENPL is negatively associated with CD8^+^ T-cell infiltration. (*P < 0.05, **P < 0.01, ***P < 0.001, ns, no significance).

**Figure 11:**
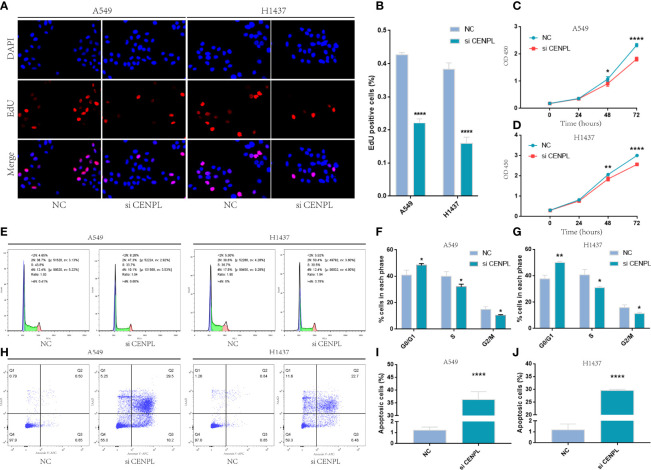
Knockdown of CENPL significantly suppressed proliferation and induced apoptosis and G0/G1 arrest in LUAD. **(A–D)** EdU and CCK8 suggest that knockdown of CENPL suppressed the proliferation of LUAD cells. **(E–G)** Knockdown of CENPL induced G0/G1 arrest of A549 and H1437 cells. **(H–J)** Knockdown of CENPL induced apoptosis of A549 and H1437 cells (*P < 0.05, **P < 0.01, ***P < 0.001).

The biological pathway analysis in **Figure 7** shows that CENPL was significantly correlated with the cell cycle in LUAD. Thus, we further detected the association between CENPL and CDK family, CDKI family, and Cyclin family expression in GEPIA2.0. The results obtained show that CENPL was positively associated with the expression of CCNA2 (R = 0.77), CCNB1 (R = 0.67), CCNB2 (R = 0.7), CCNE2 (R = 0.73), CCNF (R = 0.61), CDK1 (R = 0.67), CDK2 (R = 0.65), and CDKN3 (R = 0.72) (**Figure 9H**). Next, we designed 3 siRNA to knock down CENPL in A549 and H1437 cells. The knockdown efficiency is shown in **Figures 9C, D, G**. We found out that the knockdown of CENPL significantly decreased the expression of CDK1, CDK2, CCNA2, and CCNE2 at the mRNA level and decreased the expression of CDK2 and CCNE2 at the protein level (**Figures 10A–D**). Moreover, the results of immunohistochemistry indicated that CENPL was negatively associated with the CD8+ T-cell infiltration (**Figures 10E, F**). Next, we further detected the influence of CENPL in cell proliferation, cell cycle, and apoptosis. The results of EdU (**Figures 11A, B**) and CCK8 (**Figures 11C, D**) suggest that the knockdown of CENPL suppressed the proliferation of LUAD cells. The knockdown of CENPL significantly induced G0/G1 arrest (**Figures 11E–G**) and apoptosis (**Figures 11H–J**) of A549 and H1437 cells.

## Discussion

We found that CENPL was significantly upregulated and was correlated with the clinical stage in several cancers, and the ROC curve analysis showed that CENPL may function as a diagnostic biomarker. The prognostic analysis showed that increased CENPL was related to poorer OS in LGG, LIHC, LUAD, ACC, MESO, and PAAD. The mutation analysis showed that CENPL mutation was associated with worse OS, DFS, DSS, and PFS in breast invasive carcinoma. Among them, we found out that CENPL was significantly upregulated and correlated with the stage of LUAD. Thus, we chose LUAD to verify our bioinformatics result. In the *in vitro* experiments, CENPL was significantly upregulated in LUAD tissues and cell lines. In terms of the mechanism, the GO and KEGG analyses suggest that CENPL was correlated with cell cycle and DNA replication, and the single-cell sequence data also suggest that CENPL was positively associated with the proliferation, DNA damage, DNA repair, and cell cycle. Correlation analysis suggested a positive correlation between CENPL and CDK1, CDK2, CCNA2, CCNB1, CCNB2, CCNE2, CCNF, and CDKN3. Meanwhile, our *in vitro* experiments suggest that the knockdown of CENPL may significantly suppress the proliferation abilities of A549 and H1437 cells, and induced G0/G1 arrest and apoptosis. Furthermore, CDK2 and CCNE2 were significantly downregulated after the knockdown of CENPL.

CDK2 is a member of the CDK family and plays significant roles in cell cycle and proliferation regulation (14, 15). Houguang Liu had reported that the knockout of CDK2 may induce G0/G1 arrest and apoptosis in melanocytes (16). Moreover, Jie Yang had also reported that the downregulation of CCNE2 significantly suppressed cell proliferation and induced G0/G1 arrest (17). Thus, our studies show that the knockdown of CENPL may significantly decrease the expression of CDK2 and CCNE2, and induce G0/G1 arrest and apoptosis of A549 and H1437 cells. CENPL may function as a potential biomarker and therapy target of LUAD.

Tumor microenvironment (TME) plays important roles in cancer recurrence and drug resistance (18–20). The immune cells of TME are associated with the effect of immunotherapy, including CD4+ T cells, CD8+ T cells, MDSCs, and NKT cells (21–26). Our results suggested a positive correlation between CENPL expression and MDSC infiltration and a negative correlation between CENPL and T-cell NK infiltration in most of the cancers. As for LUAD, the obtained results show that there was a negative association between CENPL and CD8+ T cells, CD4+ T cells, T-cell NK, B cells, and myeloid dendritic cell infiltration. Furthermore, the single-cell sequence data also suggest that CENPL was negatively correlated with the inflammation level of LUAD. Our *in vitro* experiments showed a negative correlation between CENPL and CD8+ T-cell infiltration in LUAD. Thus, CENPL is related to the infiltration level of immune cells and may function as a potential immune therapy-related biomarker in LUAD.

However, some limitations still exist in our study. Although our results indicated that CENPL was associated with the immune cell infiltration of LUAD, the mechanisms are still unclear and further investigation is necessary.

## Conclusion

CENPL may function as a potential biomarker and oncogene in pan-cancer, especially LUAD. Moreover, CENPL was associated with immune cell infiltration in pan-cancer, providing a potential immune therapy target for tumor treatment.

## Data Availability Statement

The original contributions presented in the study are included in the article/supplementary material. Further inquiries can be directed to the corresponding authors.

## Ethics Statement

The studies involving human participants were reviewed and approved by Ethics Committees of the Xiangya Hospital. The patients/participants provided their written informed consent to participate in this study.

## Author Contributions

SZ and YH designed this study. ZF performed the experiments and drafted the manuscript. SZ, HS, and YH supervised the study. YC, CC, JT, PL, and YHC collected the clinical samples. All authors contributed to the article and approved the submitted version.

## Funding

This study was supported by grants from the National Key R & D Program of China (No. 2018YFC1313300), the National Natural Science Foundation of China (Nos. 81372629, 81772627, 81874073, 81974384, and 82173342), key projects from the Nature Science Foundation of Hunan Province (Nos. 2021JJ31092 and 2021JJ31048), projects from Beijing CSCO Clinical Oncology Research Foundation (Nos. Y-HR2019-0182 and Y-2019Genecast-043), and the Fundamental Research Funds of Central South University (2020zzts273 and 2019zzts797).

## Conflict of Interest

The authors declare that the research was conducted in the absence of any commercial or financial relationships that could be construed as a potential conflict of interest.

## Publisher’s Note

All claims expressed in this article are solely those of the authors and do not necessarily represent those of their affiliated organizations, or those of the publisher, the editors and the reviewers. Any product that may be evaluated in this article, or claim that may be made by its manufacturer, is not guaranteed or endorsed by the publisher.
